# Ionotropic P2X ATP Receptor Channels Mediate Purinergic Signaling in Mouse Odontoblasts

**DOI:** 10.3389/fphys.2017.00003

**Published:** 2017-01-20

**Authors:** Yuta Shiozaki, Masaki Sato, Maki Kimura, Toru Sato, Masakazu Tazaki, Yoshiyuki Shibukawa

**Affiliations:** ^1^Department of Physiology, Tokyo Dental CollegeTokyo, Japan; ^2^Department of Crown and Bridge Prosthodontics, Tokyo Dental CollegeTokyo, Japan

**Keywords:** dental pulp, dentinogenesis, odontoblasts, patch clamp, purinergic receptor

## Abstract

ATP modulates various functions in the dental pulp cells, such as intercellular communication and neurotransmission between odontoblasts and neurons, proliferation of dental pulp cells, and odontoblast differentiation. However, functional expression patterns and their biophysical properties of ionotropic ATP (P2X) receptors (P2X_1_–P2X_7_) in odontoblasts were still unclear. We examined these properties of P2X receptors in mouse odontoblasts by patch-clamp recordings. K^+^-ATP, nonselective P2X receptor agonist, induced inward currents in odontoblasts in a concentration-dependent manner. K^+^-ATP-induced currents were inhibited by P2X_4_ and P2X_7_ selective inhibitors (5-BDBD and KN62, respectively), while P2X_1_ and P2X_3_ inhibitors had no effects. P2X_7_ selective agonist (BzATP) induced inward currents dose-dependently. We could not observe P2X_1, 2/3, 3_ selective agonist (αβ-MeATP) induced currents. Amplitudes of K^+^-ATP-induced current were increased in solution without extracellular Ca^2+^, but decreased in Na^+^-free extracellular solution. In the absence of both of extracellular Na^+^ and Ca^2+^, K^+^-ATP-induced currents were completely abolished. K^+^-ATP-induced Na^+^ currents were inhibited by P2X_7_ inhibitor, while the Ca^2+^ currents were sensitive to P2X_4_ inhibitor. These results indicated that odontoblasts functionally expressed P2X_4_ and P2X_7_ receptors, which might play an important role in detecting extracellular ATP following local dental pulp injury.

## Introduction

Extracellular adenosine triphosphate (ATP) and other nucleotides play important roles in various cellular physiological and pathological functions, which are not only limited to purinergic neurotransmission for dentinal sensitivity (Shibukawa et al., 2015) or tastes (Taruno et al., 2013) but also diseases in the immune and neural systems as well as inflammatory response and pain (Burnstock, 2013), by activating plasma membrane purinergic receptors. Purinergic P2 receptors respond to extracellular nucleotides and are classified into ionotropic ATP (P2X) receptor and G protein-coupled metabotropic nucleotide (P2Y) receptor (Burnstock and Kennedy, 1985). Seven P2X receptor subunits (P2X_1_–P2X_7_) have been identified (Burnstock, 2013) and combine trimers, which form functional homo- and hetero-multimers (Burnstock, 2007). Heteromultimers of P2X_1/2_, P2X_1/4_, P2X_1/5_, P2X_2/3_, P2X_2/6_, and P2X_4/6_ have been characterized, while P2X_6_ or P2X_7_ receptors do not comprise a homomultimer or heteromultimer, respectively. P2X receptors are ATP-gated cation channels, whereas P2Y receptor subtypes are preferentially activated by nucleotides other than ATP (Abbracchio et al., 2006). All P2X receptors are cation-selective channels with almost equal permeability to Na^+^, K^+^, and significant permeability to Ca^2+^ (Jarvis and Khakh, 2009; Samways et al., 2014). It has been reported that nociceptive tooth-pulp afferent (trigeminal ganglion neurons) express P2X_3_ receptors (Cook et al., 1997) and are sufficient to elicit nociceptive behavioral responses (Adachi et al., 2010).

Odontoblasts originate from the neural crest and are located at the interface between the dentin and dental pulp. The primary function of odontoblasts is dentin formation known as dentinogenesis, during developmental, physiological, and pathological processes. In addition, recent studies have indicated that odontoblasts are sensory receptor cells for dentin sensitivity, known as the “hydrodynamic odontoblast receptor theory” (Sato et al., 2015; Shibukawa et al., 2015; Nishiyama et al., 2016) by communicating intercellularly with neurons via neurotransmitter, ATP and glutamate. Membrane deformation caused by dentinal fluid movement activates the mechanosensitive-transient receptor potential (TRP) channel; ATP is released to the extracellular space through pannexin-1, which are plasma membrane ATP-permeable channels, and activates P2X_3_ receptors on the neuron to establish neurotransmission between odontoblast and neurons (Shibukawa et al., 2015). Glutamate mediates neurotransmission between odontoblasts and metabotropic glutamate (mGlu) receptors in trigeminal ganglion neurons through glutamate-permeable anion channels (Nishiyama et al., 2016). Both ATP and glutamate also mediate intercellular odontoblast-odontoblast communication by activation of P2Y and mGlu receptors, respectively (Sato et al., 2015; Shibukawa et al., 2015; Nishiyama et al., 2016). However, these previous studies suggested that odontoblasts did not express P2X_3_ receptors, and P2X receptors could not mediate intercellular odontoblast-odontoblast communication (Sato et al., 2015). Therefore, the functional expression and the expression patterns of P2X receptors in odontoblasts have remained unclear.

To elucidate the functional expression and biophysical/pharmacological properties of P2X receptors, we measured the plasma membrane currents induced by P2X receptor activation in mouse odontoblasts.

## Materials and methods

### Solutions and reagents

A solution containing 136 mM NaCl, 5 mM KCl, 2.5 mM CaCl_2_, 10 mM HEPES, 10 mM glucose, and 12 mM NaHCO_3_ [adjusted to pH 7.4 with tris(hydroxymethyl)aminomethane] was used as a standard extracellular solution (standard ECS). To prepare a Na^+^-free solution (Na^+^-free ECS), extracellular NaCl was substituted by equimolar tetraethylammonium chloride (TEA-Cl). For the extracellular Ca^2+^-free solution and the extracellular Na^+^- and Ca^2+^-free solution (Na^+^/Ca^2+^-free solution), extracellular Ca^2+^ was simply removed (0 mM) from the standard ECS or Na^+^-free ECS, respectively.

Pharmacological agents, 5-(3-bromophenyl)-1,3-dihydro-2H-benzofuro[3,2-e]-1,4-diazepin-2-one (5-BDBD), NF110, NF449, KN62 were obtained from Tocris Bioscience (Ellisville, MO, USA). All the other reagents including adenosine 5′-triphosphate dipotassium salt dehydrate (K^+^-ATP), 2′(3′)-O-(4-Benzoylbenzoyl)adenosine 5′-triphosphate triethylammonium salt (BzATP), and αβ-methylene adenosine 5′-triphosphate (αβ-MeATP) were obtained from Sigma Aldrich Chemical Co. (St. Louis, MO, USA). Stock solutions for these agents were prepared in dimethylsulfoxide or MilliQ water (Millipore, Massachusetts, USA), and later diluted to the appropriate concentrations in either extracellular solution or culture medium. Solutions and drugs prepared in an extracellular medium were applied to the cells by a rapid solution exchanging system (Warner Instruments, Hamden, CT, USA).

### Cell culture

Mouse odontoblast lineage cells (OLCs) were cultured in an alpha-minimum essential medium containing 10% fetal bovine serum, 100 units/ml penicillin, 100 μg/ml streptomycin, and 2.5 μg/ml fungizone (Invitrogen, Carlsbad, CA, USA) at 37°C with 5% CO_2_. These cells, established through spontaneous immortalization of mouse fetal dental papilla cells upon serial passages (Arany et al., 2006), were a kind gift from Dr. Masayuki Tokuda, Kagoshima University, Kagoshima, Japan.

### Whole-cell patch-clamp recording technique

Whole-cell recordings were performed using a conventional patch-clamp recording configuration under voltage-clamp conditions. Patch pipettes (4–9 MΩ) were pulled from capillary tubes by using a DMZ universal puller (Zeitz Instruments, Martinsried, Germany), which were filled with an intracellular solution. The intracellular solution contained 140 mM KCl, 10 mM NaCl, and 10 mM HEPES (pH was adjusted to 7.2 by Tris). Whole-cell currents were measured using a patch-clamp amplifier (L/M-EPC-7+; Heka Elektronik, Lambrecht, Germany). The current traces were monitored and stored using pCLAMP (Molecular Device, Foster City, CA, USA) after digitizing the analog signals at 10 kHz (DigiData 1440A, Molecular Device) and filtering the signals digitally at 3 kHz using pCLAMP. The data were analyzed offline by using pCLAMP and the technical graphics/analysis program ORIGIN (MicroCal Software, Northampton, MA, USA). The solution temperature when measuring the whole-cell currents was maintained at 30°C.

### Calculation of the change in ionic permeability induced by P2X receptor activation

We calculated the relative change in the total ionic permeability induced by the activation of P2X receptor by using the following Equation 1:

(1) $$P_{\text{P2X}}/P_{\text{control}}=10^{\Delta \text{ErevF/2}.\text{303RT}}$$

where *P*_P2X_ is the relative total ionic permeability after the activation of P2X receptor by the agonist (BzATP or K^+^-ATP), *P*_control_ is 1.0 for the reversal potentials (E_rev_s) measured without any P2X receptor agonist in the ECS, ΔE_rev_ is the change in E_rev_ by P2X receptor agonist, *F* is Faraday's constant, *R* is gas constant, and *T* is absolute temperature. The temperature was maintained at 30°C while measuring the ramp currents.

### Statistical analysis

All data are presented as mean ± standard deviation (SD) of N observations, where N represents the number of cells tested or the number of experiments. Steel–Dwass multiple comparisons were used to determine nonparametric statistical significance. *P* < 0.05 were considered significant.

## Results

### Outwardly rectifying current in odontoblasts

**Figure 1:**
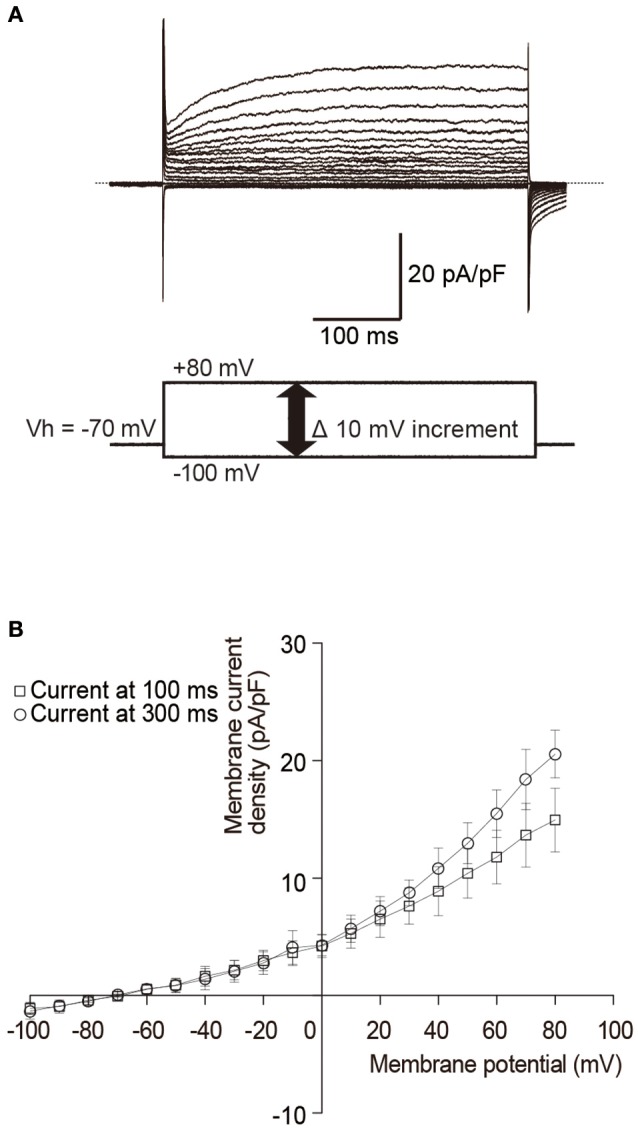
**Outwardly rectifying currents in odontoblasts. (A)** Traces show superposed whole-cell currents evoked by a sequence of 400 ms depolarizing voltage pulses at Vh of −70 mV with 10 mV increment from −100 to +90 mV. Lower panel in **(A)** shows a voltage pulse protocol. **(B)** Current-voltage (I–V) relationships of outwardly rectifying currents in odontoblasts show amplitudes of current density at 100 ms (open squares) and 300 ms (open circles) after the voltage pulse onset against the applied membrane potentials. Each point indicates the mean ± SD of three separate experiments.

Mouse odontoblast lineage cells have a cell capacitance of 32.7 pF ± 6.2 (*N* = 6). Current amplitudes were normalized to these single cell capacitance values and expressed as current densities (pA/pF). Depolarized voltage steps from −100 to +80 mV at a holding potential (Vh) of −70 mV (lower in Figure 1A) elicited outward currents (upper in Figure 1A) with a reversal potential of −61 mV (−60.3 ± 1.8; Figure 1B) in the standard ECS. These outward currents showed slow activation and non-inactivation during 400 ms depolarization pulses. The current-voltage relationship of the currents showed outward rectification with increasing membrane potentials (Figure 1B).

### K^+^-ATP-induced inward current in odontoblasts

**Figure 2:**
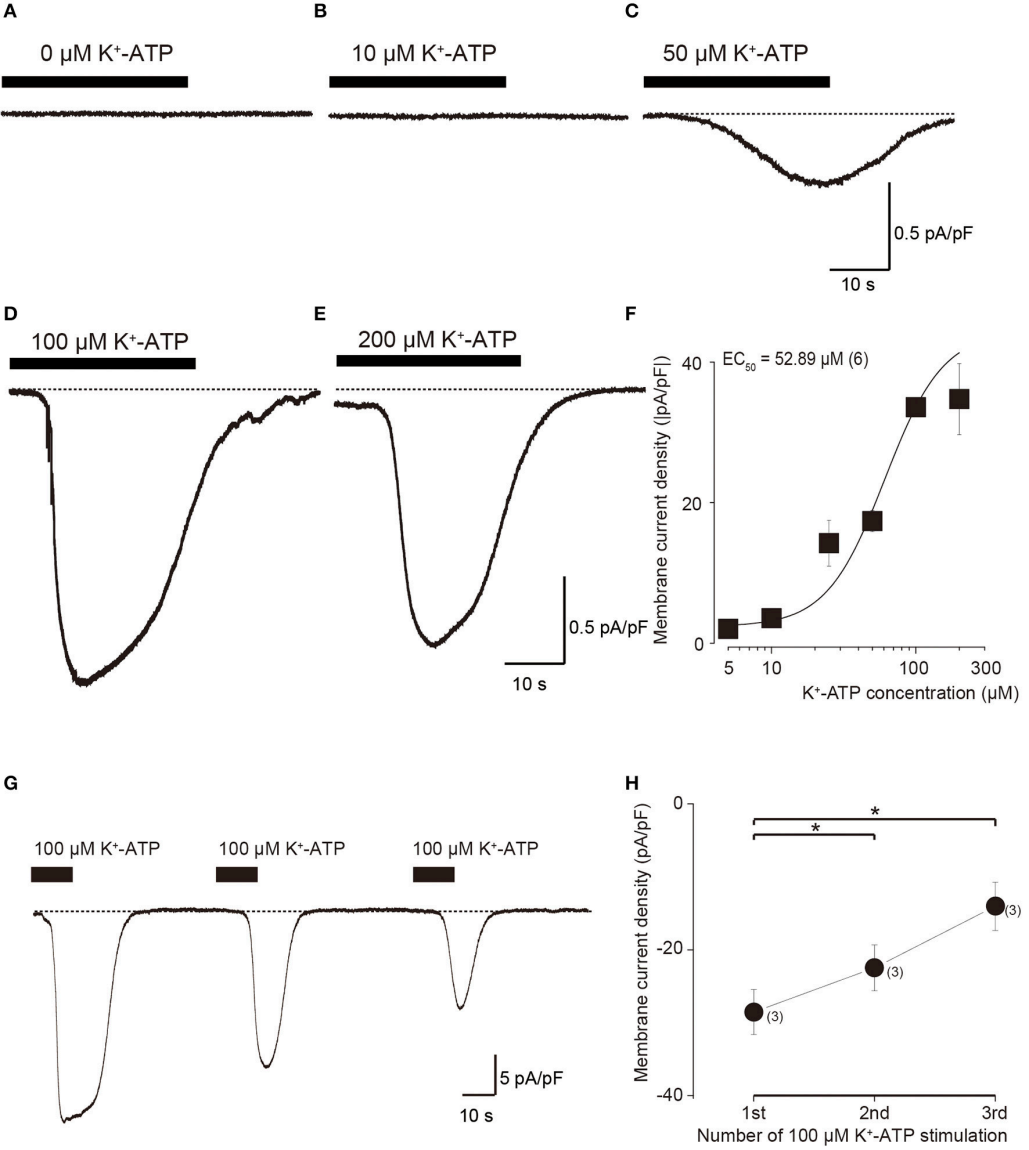
**Dose-response relationships of inward current in odontoblasts by application of various concentration of extracellular K^+^-ATP. (A–E)** Example traces of inward currents induced by various concentrations of extracellular K^+^-ATP. **(F)** Dose-response relationship between absolute values of current density induced by K^+^-ATP and their concentrations. Each data point represents the mean ± SD from six cells. The curve (solid line) on a semilogarithmic scale was fitted to the following Equation (2): $\text{I}=\left({\text{I}}_{\text{min}}-{\text{I}}_{\text{max}}\right)\;/\;1\;+{\left(\text{x}/\left(\text{K}/\text{dx}\right)\right)}^{\text{p}}+{\text{I}}_{\text{max}}$ where K is the half-maximal concentration of K^+^-ATP to activate the inward currents, I_max_ is the maximal current density and I_min_ is the minimal current density. Applied concentration of K^+^-ATP are shown by x. **(G)** Repeated application of K^+^-ATP (100 μM) induced desensitizing effect on the inward currents. An example trace of inward current elicited by three times repeated application of 100 μM K^+^-ATP with a 40-s interval with 10 s in duration at a Vh of −70 mV. **(H)** Data points show the peak current density induced by K^+^-ATP, indicating desensitizing effect. The peak current densities are shown for three successive applications of K^+^-ATP. Each data point indicates the mean ± SD of three separate experiments. Statistically significant differences between points (shown by solid lines) are indicated by asterisks, ^*^P < 0.05.

In the standard ECS, the addition of four different concentration of extracellular K^+^-ATP (10, 50, 100, and 200 μM) evoked inward currents at Vh of −70 mV, in a concentration-dependent manner (Figures 2A–E). A semilogarithmic plot (Figure 2F) illustrates membrane current densities (pA/pF) as a function of the applied concentration of extracellular K^+^-ATP, with an equilibrium binding constant (EC_50_) of 52.9 μM (*N* = 6). A series of three times of repeated applications of 100 μM K^+^-ATP (10 s in duration at 40 s intervals) elicited a significant desensitizing effect of current (Figure 2G), showing that the current amplitudes decreased with increasing times of repeated application. The amplitudes of K^+^-ATP induced current at the second and third application were significantly decreased by 78.6 ± 3.2% (*N* = 6, *P* < 0.05) and 48.9 ± 8.0% (*N* = 6, *P* < 0.05), respectively, over that at the first application (Figure 2H).

### Expression of P2X_4_ and P2X_7_ but not P2X_1_, P2X_2/3,_ and P2X_3_ receptors in odontoblasts

**Figure 3:**
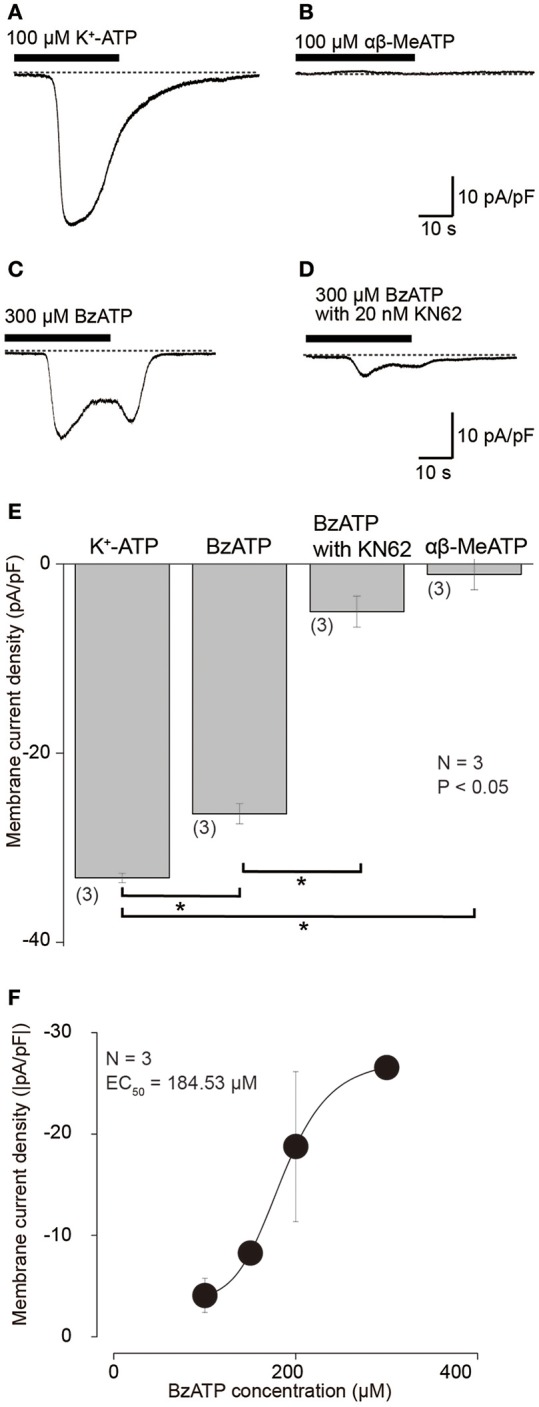
**Inward currents evoked by K^+^-ATP and BzATP, but not by αβ-MeATP. (A–D)** Example traces of inward current evoked by 100 μM K^+^-ATP **(A)** or 300 μM BzATP **(C)**. Application of 100 μM αβ-MeATP did not induce any currents **(B)**. BzATP (300 μM) induced current was suppressed by 20 nM KN62 **(D)**. These currents were recorded at Vh = −70 mV. **(E)** Bar graph shows a summary of the peak current densities elicited by application of each K^+^-ATP, BzATP, αβ-MeATP, as well as BzATP with KN62. Each bar indicates the mean ± SD of three separate experiments. Statistically significant differences between bars (shown by solid lines) are indicated by asterisks, ^*^P < 0.05. **(F)** Dose-response relationship of absolute values of current density induced by BzATP in various concentrations. Each data point represents the mean ± SD from three cells. The curve (solid line) on a semilogarithmic scale was fitted to Equation (2). Applied concentration of BzATP is shown by x.

To examine membrane expression patterns of P2X receptors in odontoblasts, we investigated the pharmacological properties of inward current induced by various extracellular purinergic stimulations. At a Vh of −70 mV, application of 100 μM K^+^-ATP induced inward currents with a peak value of 33.2 ± 0.5 pA/pF (*N* = 3; Figures 3A,E), whereas application of 100 μM αβ-MeATP, a P2X_1_, P2X_2/3_, P2X_3_ receptor agonist, could not induce any inward currents (1.1 ± 1.6 pA/pF; *N* = 3; Figures 3B,E). BzATP (300 μM), which is P2X_7_ receptor selective agonist (Salas et al., 2013; Shieh et al., 2014), evoked current with a peak value of 26.4 ± 1.1 pA/pF (*N* = 3; Figures 3C,E). In addition, BzATP-induced inward currents were inhibited by P2X_7_ receptor antagonist, 10 nM KN62 (Park et al., 2015), to the amplitudes of 5.0 ± 1.6 pA/pF (*N* = 3; Figures 3D,E). BzATP elicited inward currents in a concentration dependent manner (*N* = 3, Figure 3F).

**Figure 4:**
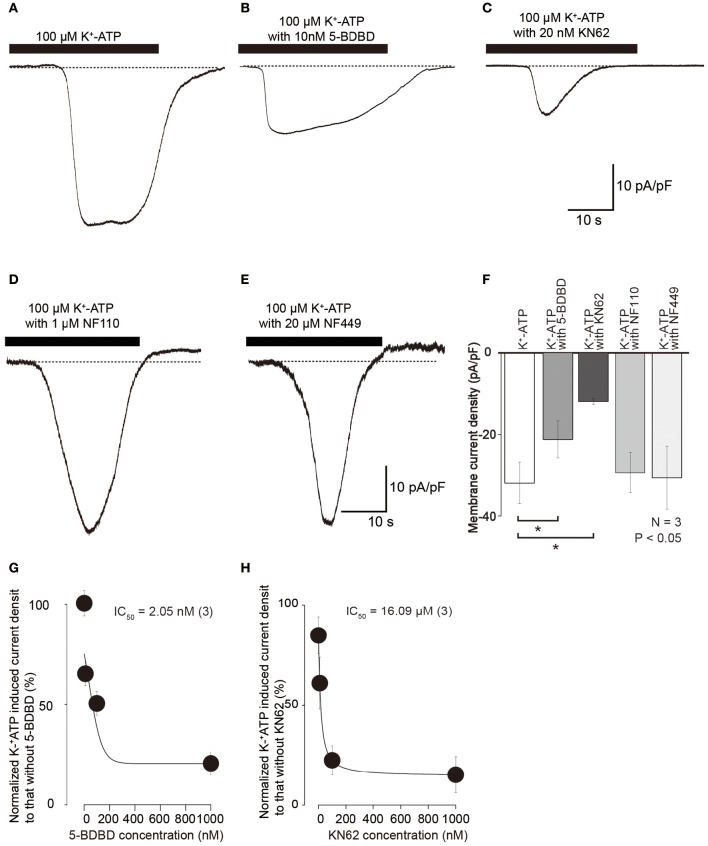
**K^+^-ATP-induced inward currents were inhibited by several selective P2X receptor agonists. (A–E)** Example traces of inward currents induced by applications of 100 μM K^+^-ATP **(A)**, 100 μM K^+^-ATP with 10 nM 5-BDBD **(B)**, 100 μM K^+^-ATP with 20 nM KN62 **(C)**, 100 μM K^+^-ATP with 1 μM NF110 **(D)**, and 100 μM K^+^-ATP with 20 μM NF449 **(E)**. **(F)** Bar graph summarizes current densities activated by 100 μM K^+^-ATP (most left) as well as 100 μM K^+^-ATP with 10 nM 5-BDBD (second left), with 20 nM KN62 (third left), with 1 μM NF110 (second right), and with 20 μM NF449 (most right). Each bar denotes the mean ± SD of three separate experiments. Statistically significant differences between bars (shown by solid lines) are indicated by asterisks, ^*^P < 0.05. Significant differences were found in the K^+^-ATP-induced currents between in the presence and absence of KN62 or 5-BDBD, while we could not observe any significant differences in the peak current density in K^+^-ATP-induced currents between in the absence or presence of NF110 or NF449. **(G,H)** Effects of 5-BDBD **(G)** and KN62 **(H)** on the absolute values of current density induced by 100 μM K^+^-ATP. Each point indicates the mean ± SD of three separate experiments. The curve (solid line) on a semilogarithmic scale was fitted to Equation (2), showing dose dependence.

Inward currents evoked by 100 μM K^+^-ATP (Figures 4A,F) were also inhibited by treatment with 10 nM 5-BDBD, a P2X_4_ antagonist (Barr et al., 2014), to 63.4 ± 12.7% (*N* = 3; Figures 4B,F) and 20 nM KN62 to 35.6 ± 1.6% (*N* = 3; Figures 4C,F). K^+^-ATP-induced inward currents were inhibited by 5-BDBD and KN62 in a concentration dependent manner (*N* = 3, Figures 4G,H). A P2X_3_ antagonist, 1 μM NF110 (Figure 4D), and P2X_1_ antagonist, 20 μM NF449 (Figure 4E), did not affect the peak amplitudes of 100 μM K^+^-ATP-induced inward currents (98.8 ± 12.2 and 98.4 ± 13.7%, respectively; Figure 4F), while these antagonists slowed activation kinetics of the inward currents. These results indicated that extracellular ATP activated inward current via P2X_4_ and P2X_7_ receptor activation. P2X_1_ and P2X_3_ receptors seem to affect the activation kinetics of ATP-induced inward currents, but did not contribute to the peak current component of the K^+^-ATP-induced inward currents.

**Figure 5:**
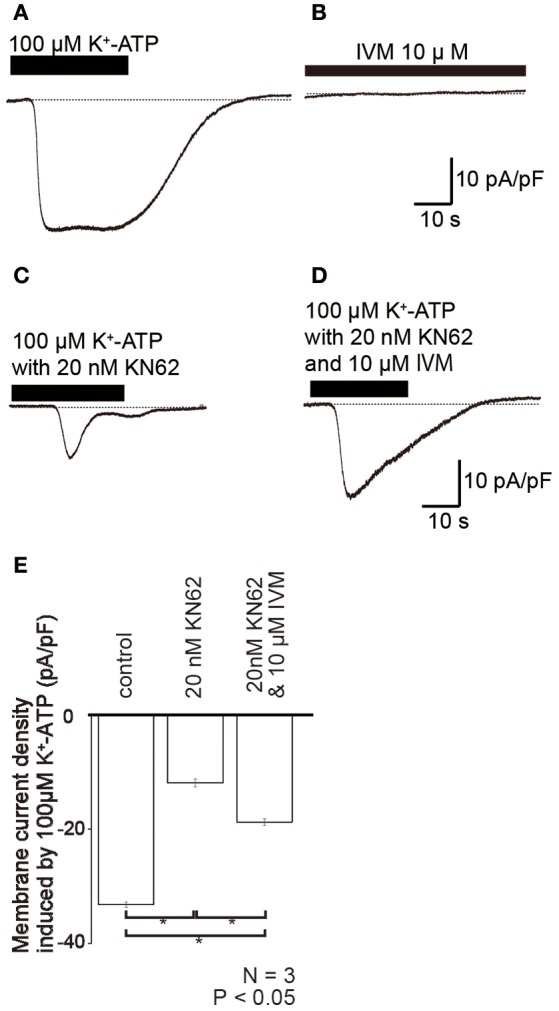
**Ivermectin (IVM), a positive selective allosteric modulator of P2X_4_ receptors, potentiated K^+^-ATP-induced inward currents. (A–D)** Example traces of inward current evoked by 100 μM K^+^-ATP **(A)**, 10 μM IVM **(B)**, 100 μM K^+^-ATP with 20 nM KN62 **(C)**, and 100 μM K^+^-ATP, and 20 nM KN62 with 10 μM IVM **(D)**. These currents were recorded at a holding potential (Vh) of −70 mV. **(E)** Bar graph summarizes the peak current densities elicited by the application of 100 μM K^+^-ATP (as control), 100 μM K^+^-ATP with 20 nM KN62 (center), and 100 μM K^+^-ATP, and 20 nM KN62 with 10 μM IVM (right). Each bar indicates the mean ± standard deviation (SD) of three separate experiments. Statistically significant differences between bars (shown by solid lines) are indicated by asterisks, ^*^P < 0.05.

K^+^-ATP-induced inward currents (Figures 5A,E) were suppressed by P2X_7_ receptor antagonist (20 nM KN62; Figures 5C,E). However, in presence of 10 μM ivermectin (IVM), a positive selective allosteric modulator of P2X_4_ receptors (Sim et al., 2007), the amplitude of residual K^+^-ATP-induced inward current component (Figure 5D) increased to 158.1 ± 5.8% (*N* = 3; Figure 5E). Ivermectin failed to induce currents in the absence of any P2X agonists (Figure 5B).

### Cation conductance and Ca^2+^ block of ATP-induced currents

**Figure 6:**
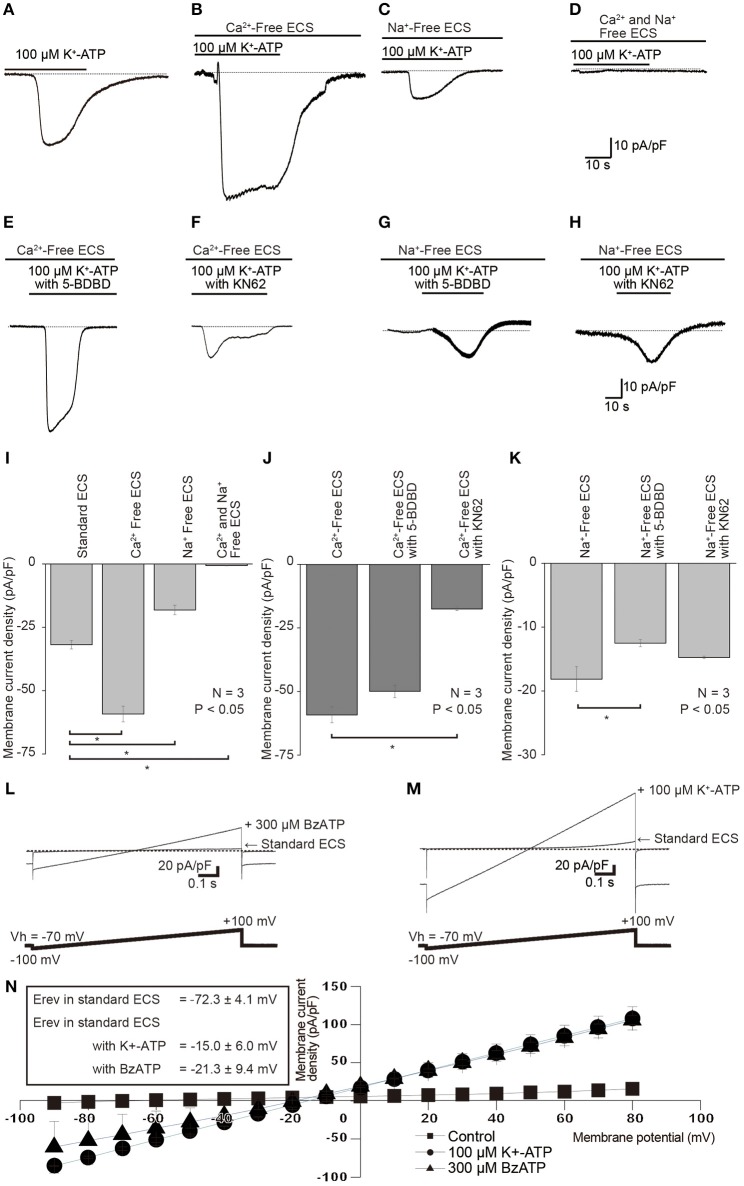
**Effects of removals of extracellular cations, as well as of P2X receptor antagonist on each K^+^-ATP-induced Na^+^ or Ca^2+^ current. (A–D)** Example traces of K^+^-ATP-induced inward currents are shown, which were obtained in the standard ECS **(A)**, Ca^2+^-free ECS **(B)**, Na^+^-free ECS **(C)**, or Ca^2+^ and Na^+^-free ECS **(D)**. **(E,F)** Example traces of K^+^-ATP (100 μM) induced currents that were recorded in the Ca^2+^-Free ECS with 10 nM 5-BDBD **(E)** or 20 nM KN62 **(F)**. **(G,H)** Example traces of K^+^-ATP (100 μM) induced inward current, which were recorded in Na^+^-free ECS with 10 nM 5-BDBD **(G)** or 20 nM KN62 **(H)**. **(I)** Bar graph shows the peak current densities evoked by 100 μM K^+^-ATP in the standard ECS, Ca^2+^-Free ECS, Na^+^-Free ECS, or Ca^2+^- and Na^+^-Free ECS. **(J)** Summary bar graph shows the peak current densities evoked by 100 μM K^+^-ATP in Ca^2+^ free ECS without or with 20 nM KN62 and 10 nM 5-BDBD. **(K)** Summary bar graph shows the peak current densities evoked by 100 μM K^+^-ATP in Na^+^ free ECS without or with 20 nM KN62 and 10 nM 5-BDBD. Each bar denotes the mean ± SD of three separate experiments. Statistically significant differences between bars (shown by solid lines) are indicated by asterisks, ^*^P < 0.05. **(L,M)** I–V relationships of the currents induced by P2X receptor agonists. Voltage-ramp protocol from −100 to +100 mV (0.2 mV/ms) at a Vh of −70 mV was applied to the cells (bottoms in **L**,**M**). Traces show the example currents, which were recorded in standard ECS and in standard ECS with 300 μM BzATP **(L)** or 100 μM K^+^-ATP **(M)**. **(N)** I–V relationships for ramp current without (closed squares) or with 100 μM K^+^-ATP (closed circles) and 300 μM BzATP (closed triangles). The data points show the mean ± SD of three separate experiments. Reversal potential (E_rev_) for the currents were −72.3 ± 4.1 mV in standard ECS, −15.0 ± 6.0 mV in the ECS with K^+^-ATP, or −21.3 ± 9.4 mV in standard ECS with BzATP.

When we removed extracellular Ca^2+^ from standard ECS (Ca^2+^-free solution), K^+^-ATP-induced peak inward current amplitudes (Figures 6A,I) increased to 177.7 ± 13.7% (*N* = 3; Figures 6B,I), as compared to the current amplitudes with standard ECS. When we perfused Na^+^-free ECS (but with presence of extracellular Ca^2+^), 100 μM K^+^-ATP-evoked currents decreased their peak amplitudes to 46.0 ± 9.2% (*N* = 3; Figures 6C,I). In the absence of both extracellular Ca^2+^ and Na^+^ in the extracellular solution (Na^+^ and Ca^2+^-free ECS), we could record only a residual small current component (0.6 ± 0.2 pA/pF, *N* = 3; Figures 6D,I) by an application of 100 μM K^+^-ATP. In the absence of extracellular Ca^2+^, the amplitudes of extracellular K^+^-ATP (100 μM)-evoked Na^+^ currents were slightly inhibited by application of 20 nM 5-BDBD to 84.7 ± 9.3% (*N* = 3; Figures 6E,J), and significantly reduced by 10 nM KN62 to 29.9 ± 4.7% (*N* = 3; Figures 6F,J). 5-BDBD did not induce any significant inhibitory effect on the K^+^-ATP-evoked Na^+^ currents (Figure 6J). In the absence of extracellular Na^+^, K^+^-ATP-induced Ca^2+^ currents were significantly inhibited by 20 nM 5-BDBD (69.9 ± 8.8%, *N* = 3; Figures 6G,K), but slightly affected by 10 nM KN62 (83.1 ± 15.0%, *N* = 3; Figures 6H,K). KN62 did not elicit any significant inhibition on the K^+^-ATP-evoked Ca^2+^ currents.

To further analyze the changes in the total ionic permeability induced by P2X receptor activation, we recorded ramp currents to determine the current-voltage (I–V) relationship and analyze E_rev_s during application of P2X receptor agonists. When we applied a voltage-ramp protocol from −100 to +100 mV (0.2 mV/ms) at a Vh of −70 mV (upper, Figures 6L,M), outwardly rectifying currents were observed in standard ECS (Figures 6L–N). Applications of 300 μM BzATP (Figure 6L) and 100 μM K^+^-ATP (Figure 6M) increased both inward and outward current components with changes in E_rev_s. E_rev_s of the ramp currents were shifted +57 mV toward depolarizing potentials by 100 μM K^+^-ATP and +51 mV to them by 300 μM BzATP, on comparing Erevs obtained without K^+^-ATP and BzATP in standard ECS (*N* = 3, Figure 6N). We calculated the relative changes in the total ionic permeability induced by P2X receptor activations using Equation (1). The relative *P*_P2X_ was 7.1 for BzATP-induced current and 8.8 for K^+^-ATP-induced one.

## Discussion

The OLCs used in this study are positive for various odontoblast-representative transcripts such as dentin sialophosphoprotein, dentin matrix protein-1, and nestin (Arany et al., 2006; Sato et al., 2013). In the present study, BzATP, a selective agonist of P2X_7_ receptor, induced inward current in odontoblasts. Not only BzATP-induced currents but also K^+^-ATP-induced currents were sensitive to KN62, a selective antagonist for P2X_7_ receptors. In addition, the amplitudes of K^+^-ATP-induced currents were suppressed by 5-BDBD, a selective P2X_4_ receptor inhibitor (Gofman et al., 2014). On the contrary, αβ-MeATP (an agonist for P2X_1_, P2X_2/3_, and P2X_3_ as well as for P2X_4_ and P2X_4/6_ receptors) failed to induce any inward currents in odontoblasts at 100 μM concentration. Studies show that 10 μM αβ-MeATP activates P2X_1_, P2X_2/3_, P2X_3_, and P2X_4/6_ subtypes, while P2X_4_ receptors are less sensitive to αβ-MeATP action (Khakh et al., 2001; Jarvis and Khakh, 2009). Furthermore, the P2X_3_ and P2X_1_ antagonist NF110 and NF449, respectively, failed to induce any inhibitory effect on the K^+^-ATP-induced currents. The P2X_7_ receptor inhibitor KN62 suppressed the K^+^-ATP-induced inward current. However, in presence of IVM the amplitude of residual K^+^-ATP-induced inward current increased. These results indicate that odontoblasts express P2X_4_ and P2X_7_ but not P2X_1_, P2X_2/3_, and P2X_3_ receptors. In addition, the expression of P2X_4/6_ heteromer in odontoblasts is implausible (Jarvis and Khakh, 2009).

In our previous study, we have shown that ATP, as intercellular-/neuro-transmitter, which was released from mechanically stimulated odontoblasts increased the intracellular Ca^2+^ concentration in odontoblasts as well as neurons located nearby the stimulated odontoblasts (Sato et al., 2015; Shibukawa et al., 2015; Nishiyama et al., 2016). An application of P2X_3_ receptor antagonist did not elicit mechanical stimulation-induced response in nearby odontoblasts, but did in the neurons (Shibukawa et al., 2015). These results were in line with the present results showing the implication of the lack of P2X_3_ receptor in odontoblasts, while the P2X_3_ receptor in the TG neurons plays an important role in the sensory transduction sequence by receiving ATP from mechanically stimulated odontoblasts (Shibukawa et al., 2015). We could not obtain the results showing whether the P2X_2_, P2X_5_, and P2X_6_ are expressed functionally in odontoblasts or not in this study, since there are no commercially available selective pharmacological ligands for these P2X receptor subtypes to date. Although, we showed that odontoblasts exhibit functional expression of P2X_4_ and P2X_7_ receptors but not P2X_1_, P2X_2/3_, P2X_3_, and P2X_4/6_, further study is warranted to throw light on the expression of P2X_2_, P2X_5_, and P2X_6_ (homomer) receptors.

The peak amplitudes of K^+^-ATP-induced currents were increased by the removal of Ca^2+^, whereas they were decreased by the removal of Na^+^ from the extracellular medium. In addition, K^+^-ATP-induced currents were almost completely abolished in the Na^+^ and Ca^2+^-free ECS. Thus, the K^+^-ATP induced current was composed by Na^+^ and Ca^2+^ conductance. It has been well-known that P2X receptors show relative high Ca^2+^ permeability (Samways et al., 2014). In this study, K^+^-ATP-induced Ca^2+^ currents in the Na^+^-Free ECS were significantly inhibited by P2X_4_ receptor antagonist (5-BDBD), while not by P2X_7_ receptor antagonist (KN62), indicating that P2X_4_ receptor in odontoblast has high Ca^2+^ permeability. These results are also in line with the results showing that P2X_4_ receptor showed highest relative Ca^2+^ permeability among the P2X family (Egan and Khakh, 2004). The cation permeability for P2X_7_ receptor remains debatable, which also shows high Ca^2+^ permeability; however, the Ca^2+^ curries ~5% of the total inward current through P2X_7_ (Jarvis and Khakh, 2009; Samways et al., 2014). It has been known that the cation permeability by P2X receptor activation were modulated by divalent cations as “Ca^2+^ dependent block” (Jarvis and Khakh, 2009; Kasuya et al., 2016), in which the ion permeability of P2X receptors are inhibited by the extracellular Ca^2+^ in a concentration-dependent manner. The Ca^2+^ dependent block for P2X receptors was also well-described in P2X_7_ receptors (Yan et al., 2011; Liang et al., 2015). When we removed extracellular Ca^2+^ from the standard ECS (Ca^2+^-free ECS), the amplitude of K^+^-ATP-induced currents augmented compared to those in the standard ECS. These K^+^-ATP-induced Na^+^ currents were strongly inhibited by an antagonist of P2X_7_ receptors, but not of P2X_4_ receptors. Thus, the results indicated that the ionic permeability of P2X_7_ receptors in odontoblasts were also blocked by extracellular Ca^2+^, showing Ca^2+^ dependent block. In addition, the main ionic component of cation permeability for P2X_4_ receptor was Ca^2+^, whereas that for P2X_7_ was Na^+^. We could observe each residual K^+^-ATP-induced Ca^2^ and Na^+^ current component after P2X_4_ and P2X_7_ receptor inhibition. These were carried by other P2X receptor activation, including P2X_2_, P2X_5_, and P2X_6_ receptors; however, further study will be needed.

In previous studies, we showed that P2X receptors, as well as ionotropic glutamate receptors, could not mediate intercellular odontoblast-odontoblast communication (Sato et al., 2015; Nishiyama et al., 2016). We could hardly record evoked-inward currents [that were activated by intercellular mediator(s)] in the odontoblast located 5 μm away from the mechanically stimulated odontoblast (Sato et al., 2015). These previous results had implied that the ionotropic receptor [such as ionotropic ATP (P2Xs) and/or glutamate receptors] activation by released intercellular transmitters—not only of ATP but also of glutamate—following mechanical stimulation of the odontoblasts, are hardly involved in the inter-odontoblast communication. In the present study, the EC_50_ of K^+^-ATP on the inward currents in odontoblasts was 52.9 μM. Comparing the EC_50_ of ATP for P2X_1_–P2X_6_ receptor activation, this value was of 5–50 times higher concentration than those reported by other studies, in which they have reported EC_50_ ranging from 1.0 to 10 μM (Jarvis and Khakh, 2009). The value of EC_50_ of K^+^-ATP in this study was in line with that for EC_50_ of ATP for P2X_7_ receptor activation (100 μM: Khakh et al., 2001). Recently, the concentration of released ATP by external dentin cold-stimulation has been reported to be “nM” range in an *in vitro* human tooth perfusion model (Egbuniwe et al., 2014; Liu et al., 2015). Based on our results, P2X receptor subtypes expressed in odontoblasts need ~1000 times as high of a concentration of extracellular ATP to be activated (ca. 50–100 μM range), as compared to the ATP concentration by dentin stimulation induced releases. Therefore, by the existence of the differences in the affinity of ATP, P2X receptors in odontoblasts seem not to mediate intercellular odontoblast-odontoblast communication (Sato et al., 2015; Shibukawa et al., 2015), while P2X receptors in neurons mediate neurotransmission between odontoblasts and neurons for sensory transduction sequence for the dentinal pain (Kuroda et al., 2012; Shibukawa et al., 2015).

On the other hand, ATP that leaked from injured cells in dental pulp could activate P2X receptors in odontoblasts. The intracellular concentration of ATP is in the mM range (Imamura et al., 2009). Following tissue injury, the increase in the local concentration of ATP at the injured site reached the sub-mM range, which such high enough concentration of ATP might be capable to activate P2X receptors in odontoblasts. Thus, we imply that P2X receptors in odontoblasts play an important role in the biophylaxis function for the dental pulp to detect local tissue injury, rather than intercellular odontoblast communication.

In conclusion, odontoblasts functionally expressed P2X receptor subtypes of P2X_4_ and P2X_7_ receptors, but not P2X_1_, P2X_2/3_, P2X_3_, and P2X_4/6_ receptors. P2X_4_ receptors have high Ca^2+^ permeability, whereas P2X_7_ receptors have Na^+^ conductance with a Ca^2+^ dependent block.

## Author contributions

Yuta S carried out the measurement membrane currents. Yoshiyuki S, TS, and MT participated with design of the study. Yuta S and Yoshiyuki S performed the statistical analysis. Yoshiyuki S conceived of the study, and participated in its design and coordination and helped to draft the manuscript. All authors read and approved the final manuscript.

### Conflict of interest statement

The authors declare that the research was conducted in the absence of any commercial or financial relationships that could be construed as a potential conflict of interest.
